# Temperature and cardiovascular diseases: exploring associations in India and public health insights: Shreya Shrikhande

**DOI:** 10.1093/eurpub/ckac131.157

**Published:** 2022-10-25

**Authors:** S Shrikhande, H Pedder, M Roosli, MA Dalvie, L Ravivarman, A Gasparrini, J Utzinger, G Cisse

**Affiliations:** 1 Ecosystem Health Sciences, Swiss Tropical and Public Health Institute, Allschwil, Switzerland; 2 Faculty of Science, University of Basel, Basel, Switzerland; 3 Population Health Sciences, University of Bristol, Bristol, UK; 4 Environmental and Occupational Health Research, School of Public Health and Family Medicine, University of Cape Town, Cape Town, South Africa; 5 Department of Health and Family Welfare Services, Government of Puducherry, Puducherry, India; 6 Centre for Statistical Methodology, LSHTM, London, UK

## Abstract

**Background:**

Climate change has far-reaching consequences on human health globally. Cardiovascular diseases (CVDs), the global leading cause of death, are climate sensitive, mainly to temperature. The temperature-CVD association is region-specific, with several studies from Europe but relatively few from low-and-middle-income countries (LMICs).

**Methods:**

We used a binomial regression model to analyze the association between apparent temperature and in-hospital CVD mortality in Puducherry city. A distributed lag non-linear model was used to capture the delayed and non-linear trends over a 21 day lag period to estimate the burden of in-hospital CVD mortalities attributable to non-optimal temperature between 2010 and 2020.

**Results:**

Tapp in Puducherry ranges from 23°C to 40°C. We found that the optimal temperature range for Puducherry is between 33°C and 35°C with respect to CVDs. Temperatures both above and below the optimal temperature range were associated with an increased risk of overall in-hospital CVD mortalities, resulting in a U-shaped association curve. Up to 20% of the CVD deaths could be attributable to non-optimal temperatures, with a slightly higher burden attributable to cold (11.2%) than heat (9.12%). We also found that males above 60 years of age were more vulnerable to colder temperatures while females above 60 years were more vulnerable to the heat. Mortality with cerebrovascular accidents was associated more with heat compared to cold, and ischemic heart diseases did not seem to be affected by temperature.

**Conclusions:**

Both cold and heat is associated with CVD mortality in Puducherry. The comparison of the results of this exploratory Indian study with those from European contexts show that the associations differ based on several factors. There are also age, gender and CVD type differences in Tapp attributable CVD mortalities. More region specific studies on Tapp- CVD mortality are needed from LMICs to better understand this association and build capacity.

**Key messages:**

• The regional burden of cold attributable CVD deaths needs to be considered along with heat. Age and gender specific differences in the association need to be further studied globally.

• The development regional and contextual climate-health action plans, as seen in some European countries, could be enhanced by such studies and reduce the burden of temperature attributable CVD deaths.

